# Genomic prediction for sugarcane diseases including hybrid Bayesian-machine learning approaches

**DOI:** 10.3389/fpls.2024.1398903

**Published:** 2024-05-01

**Authors:** Chensong Chen, Shamsul A. Bhuiyan, Elizabeth Ross, Owen Powell, Eric Dinglasan, Xianming Wei, Felicity Atkin, Emily Deomano, Ben Hayes

**Affiliations:** ^1^ Center for Animal Science, The Queensland Alliance for Agriculture and Food Innovation, The University of Queensland, Brisbane, QLD, Australia; ^2^ Sugar Research Australia, Woodford, QLD, Australia; ^3^ Queensland Micro- and Nanotechnology Centre, Griffith University, Nathan, QLD, Australia; ^4^ Center for Crop Science, The Queensland Alliance for Agriculture and Food Innovation, The University of Queensland, Brisbane, QLD, Australia; ^5^ Sugar Research Australia, Indooroopilly, QLD, Australia

**Keywords:** genomic prediction (GP), deep learning, Bayesian alphabet, GBLUP, sugarcane disease

## Abstract

Sugarcane smut and Pachymetra root rots are two serious diseases of sugarcane, with susceptible infected crops losing over 30% of yield. A heritable component to both diseases has been demonstrated, suggesting selection could improve disease resistance. Genomic selection could accelerate gains even further, enabling early selection of resistant seedlings for breeding and clonal propagation. In this study we evaluated four types of algorithms for genomic predictions of clonal performance for disease resistance. These algorithms were: Genomic best linear unbiased prediction (GBLUP), including extensions to model dominance and epistasis, Bayesian methods including BayesC and BayesR, Machine learning methods including random forest, multilayer perceptron (MLP), modified convolutional neural network (CNN) and attention networks designed to capture epistasis across the genome-wide markers. Simple hybrid methods, that first used BayesR/GWAS to identify a subset of 1000 markers with moderate to large marginal additive effects, then used attention networks to derive predictions from these effects and their interactions, were also developed and evaluated. The hypothesis for this approach was that using a subset of markers more likely to have an effect would enable better estimation of interaction effects than when there were an extremely large number of possible interactions, especially with our limited data set size. To evaluate the methods, we applied both random five-fold cross-validation and a structured PCA based cross-validation that separated 4702 sugarcane clones (that had disease phenotypes and genotyped for 26k genome wide SNP markers) by genomic relationship. The Bayesian methods (BayesR and BayesC) gave the highest accuracy of prediction, followed closely by hybrid methods with attention networks. The hybrid methods with attention networks gave the lowest variation in accuracy of prediction across validation folds (and lowest MSE), which may be a criteria worth considering in practical breeding programs. This suggests that hybrid methods incorporating the attention mechanism could be useful for genomic prediction of clonal performance, particularly where non-additive effects may be important.

## 1 Introduction

Sugarcane smut caused by fungus, *Sporisorium scitamineum is* a major disease affecting sugar cane in Australia and some other countries causing yield losses of 40% to 60% in susceptible varieties (Hoy et al., 1986; Bhuiyan et al., 2022). Pachymetra root rot is another serious fungal disease, caused by *Pachymetra chaunorhiza*, can cause 40% yield reduction in susceptible variety (Magarey, 1994). The narrow sense heritability for smut ranges from 0.47 to 0.55 and 0.22 to 0.65 for Pachymetra root rot (Wu et al., 1988; Croft and Berding, 1994). These moderate to high heritabilities suggest that there is a substantial potential for improved resistance using selection based on estimated breeding values for resistance against both diseases.

Genomic selection is a technology to improve genetic gain by utilising genome-wide markers to capture mutations of small effect that typically underpin variation in complex traits, and has been widely applied to plant breeding (Meuwissen et al., 2001; Goddard and Hayes, 2007; Heffner et al., 2009). Linear mixed models, such as best linear unbiased prediction (BLUP) including GBLUP and SNP BLUP have been widely used in genomic predictions (Goddard, 2009; Endelman, 2011; Su et al., 2012; Clark and Van Der Werf, 2013; Beyene et al., 2021). BLUP models assume all SNP would have a very small but non-zero effects and follow a normal distribution. BLUP models generally perform well across a wide range of species and traits (Habier et al., 2013).

Other algorithms for genomic prediction have been proposed that have at least two possible theoretical advantages over BLUP models, particularly for disease traits in polyploid crops. Bayesian models such as BayesC (Habier et al., 2011) and BayesR (Erbe et al., 2012),allow a proportion of markers to have a moderate or large effect and may have an advantage as some mutations of large effects have been reported for crop disease resistance, for example in wheat (Thambugala et al., 2020; Su et al., 2021), potatoes (Sobkowiak et al., 2022) and peanuts (De Blas et al., 2021). Secondly, other methods may capture non-additive effects caused by either gene-by-gene interactions or interaction between ploidies (Wu et al., 1992). For example, BLUP can be extended to include non-additive effects, such as dominant effect and epistatic effects (caused by marker interactions) (Vitezica et al., 2017). Extended GBLUP models found substantial dominance and particularly epistatic variation for yield in sugarcane (Yadav et al., 2021).

Genomic prediction using Machine Learning (ML) algorithms has been investigated across a range of species and traits. Ensemble algorithms such as random forest and boosting have been shown to have good performance in crops and animal breeding, as have some variants of neural networks (Vanraden, 2008; Heslot et al., 2012; Blondel et al., 2015; Abdollahi-Arpanahi et al., 2020). Deep learning (DL) uses complex structures in which one predictor could be learned by multiple neural weights, and flexible tunning algorithms which would also be useful to maximize DL learning ability. Up to now, ML methods including some standard DL models have been demonstrated to have comparable performance with linear models in some cases, however there is currently no universally outstanding ML approach that performs consistently well across the wide range cases of genomic prediction where they have been evaluated (Ma et al., 2018; Abdollahi-Arpanahi et al., 2020; Zingaretti et al., 2020; Montesinos-López et al., 2021).

The attention mechanism (Self-attention), the core theory of the transformer, is suggested to have capacity to capture sequence-wide positions within inputs of sequence features (words, signals, pixels, etc) in order to determine “end-to-end representation” of the sequence (Vaswani et al., 2017). For genomic prediction, such structures could bring potential benefits in capturing marker-by-marker interactions. In addition, unlike MLP or CNN which would normally reshape the input data, a sequence of features passed into an attention mechanism could retain more information, which could makes it more straightforward to understand individual marker contributions in model interpretations (Zeng et al., 2022; Katz and Belinkov, 2023).

One challenge with using attention networks and other ML methods for genomic prediction is the very large number of parameters that must be estimated, often from modest datasets. With the tens of thousands of markers commonly used in genomic prediction, the number of parameters in DL methods with multiple layers and attention networks may number in the millions, with just a few thousand phenotype observations. One possibility for such methods is to cut down the number of markers, based on their marginal additive effects, and use just these in the ML methods. The reduction in number of markers from genome wide association studies (GWAS) to retain only significant markers has been applied using both simulated and real datasets, and could enable more accurate estimates of interaction effects (Maciukiewicz et al., 2018; Abdollahi-Arpanahi et al., 2020). Here we propose hybrid models, using combinations of Bayesian alphabet models or GWAS to select subsets of markers which are passed to ML methods for genomic prediction of target traits. High confidence markers can be selected by posterior inclusion probability (estimated via Bayesian approaches) or p-values (estimated via GWAS). ML models would use these marker subsets to perform genomic prediction, ideally with better estimates of interactions and reduced risks of overfitting.

Our aim was to evaluate and compare four major types of algorithms, including GBLUP, Bayesian alphabets, ML methods (including attention networks), and hybrid models, for prediction accuracy of clonal performance for two important sugarcane diseases: smut and Pachymetra.

## 2 Materials and methods

### 2.1 Genotyping and phenotyping

There were 4702 and 1988 clones phenotyped and genotyped for smut and Pachymetra root rot respectively, based on number of sugarcane clones that were tested in the trials for each disease. There was a single trial for each disease, in a single year. Genotyping was performed on the SRA/CSIRO array, with 26,086 markers passing quality control (at least 90% of clones genotyped with high QC score for each SNP). Genotypes were formatted as diploid genotypes including AA (2), AT (1) and TT (0) following (Aitken et al., 2016). The very small proportion of missing values for genotypes (approximately 1%) were imputed by sampling based on allele frequency.

The raw phenotype, disease infection score, was rescaled into BLUPs via mixed models aimed to remove experimental designing effects. These disease infection BLUPs were then scaled into ordinal disease rating scores from 1-9 (where 1 = resistant, 9 = susceptible) as phenotypes, following characterising procedure described in Hutchinson and Daniels (1972). Ordinal disease rating scores were treated as continuous values, inspection revealed a very approximately normal distribution of scores for each trait. It should be pointed out that no pedigree information was involved during the generation of phenotypic BLUPs.

### 2.2 Genomic best linear unbiased prediction

An additive GBLUP and extended-GBLUP model were fitted to the data, the later, included dominance and epistatic random effects as well as additive effects (as described in Yadav et al., 2021).


(1) y= u + Za+ Zd+ Ze+ ϵ

(2) $${\text{A}}_{\text{jk}}=\;\frac{1}{\text{N}}\sum_{\text{i}=1}^{\text{N}}\frac{\left({\text{x}}_{\text{ij}}-2{\text{p}}_{\text{i}}\right)\left({\text{x}}_{\text{ik}}-2{\text{p}}_{\text{i}}\right)}{2{\text{p}}_{\text{i}}\left(1-{\text{p}}_{\text{i}}\right)}$$

(3) $${\text{D}}_{\text{jk}}=\;\frac{1}{\text{N}}\sum_{\text{i}=1}^{\text{N}}\frac{\left({\text{x}}_{\text{ij}}-2{\text{p}}_{\text{i}}^{2}\right)\left({\text{x}}_{\text{ik}}-2{\text{p}}_{\text{i}}^{2}\right)}{2{\text{p}}_{\text{i}}^{2}\left(1-{\text{p}}_{\text{i}}^{2}\right)}$$

Where **y** was the vector of phenotypes (disease rating scores), with one element for each clone measured, **Z** was the design matrix allocating records to clones, and **a**, **d** and **e** are the vectors of genetic values for the random additive component, dominance component and epistatic component respectively, and **ϵ** is a random error term, with one element for each vector for each clone (Equation 1).

Genomic relationship matrices among clones for additive and dominance effects (Yang et al., 2011; Zhu et al., 2015) were constructed as described in Equations 2, 3 were computed via program “GCTA”, version 1.94 (Yang et al., 2011). The epistatic relationship matrix was calculated by taking the Hadamard product of the additive relationship matrix (E= A∘A) (Cockerham, 1954; Jiang and Reif, 2020). Residual, additive, dominance and epistatic effects were assumed to be normally distributed, $\epsilon \sim \;N\left(0,\;I\sigma_{\epsilon }^{2}\right)$ , where $\sigma_{\epsilon }^{2}$ , is the residual variance. $a\;\sim \;N\left(0,\;\sigma_{A}^{2}\right)$ , where $\sigma_{A}^{2}$ is the additive genetic variance captured by SNPs, $d\sim \;N\left(0,\;D\sigma_{D}^{2}\right)$ , where ***D*** is the dominance relationship matrix as described above and $\sigma_{D}^{2}$ is the dominance variance. $e\;\sim \;N\left(0,\;G_{AA}\sigma_{E}^{2}\right)$ , where $\sigma_{E}^{2}$ is the epistatic variance. Variance components (additive, dominance, epistatic and error variances) were estimated with GREML using MTG2, version 2.22 (Lee and Van Der Werf, 2016).

### 2.3 BayesC and BayesR

The Bayesian approaches used in this study (BayesC and BayesR) used a model that fits all SNP effects as random (Equation 4);


(4) y=1μ+Xg+e

where ***y*** is the observed disease rating scores, ***μ*** is the mean, 1 ***μ*** is a vector of ones, ***X*** is the SNP genotype matrix, ***g*** is the vector of SNP effects and ***e*** is a vector of random residuals.

BayesC (Habier et al., 2011) has the assumption that SNPs can have zero or non-zero (additive) effects on the trait, with non-zero effects following a normal distribution:


$$non-zero\;with\;prior\;probability\;\pi :g_{i}\;∼\;N\left(0,\sigma^{2}\right)$$

$$zero\;with\;prior\;probability\;\left(1-\pi \right):g_{i}=0$$

BayesR assumes SNPs have effects that are either zero, derived from a normal distribution with very small variance, derived from a distribution with small variance, or derived from a normal distribution with moderate variance (Erbe et al., 2012). So $g\sim N\left(0,\sigma_{i}^{2}\right)$ with four possibilities for $\sigma_{i}^{2}=\left\{0,\;0.01*\sigma_{g}^{2},0.1*\sigma_{g}^{2},1*\sigma_{g}^{2}\right\}$ , where $\;\sigma_{g}^{2}$ is the genetic variance of the trait. So each SNP effect is from one of four possible normal distributions: $\;N(0,0*\sigma_{g}^{2}$ ), $N(0,0.01*\sigma_{g}^{2})$ , $N(0,0.1*\sigma_{g}^{2})$ , and $N(0,1*\sigma_{g}^{2})$ . As described by Erbe et al. (2012), there are two latent parameters in the BayesR model, b(i,k) and **Pr**. ***b*** (***i***, ***k***) defines whether the SNP ***i*** follows normal distribution ***k*** (***k*** = 1,2,3,4), with


(5) $$p\left({\text{g}}_{\text{i}}|\text{b}\left(\text{i},\text{k}\right)\right)=\{\begin{array}{c} 0,\;\;\;\;\;\;\;\;\;\;\;\;\;\text{b}\left(\text{i},1\right)=1\;\\ \frac{1}{\sqrt{2\text{π}{\text{σ}}_{\text{i}}^{2}\left[k\right]}}\text{exp}\left(-\frac{{\text{g}}_{\text{i}}^{2}}{2{\text{σ}}_{\text{i}}^{2}\left[\text{k}\right]}\right),\;\;\;\;\text{b}\left(\text{i},\text{k}\right)=1\left(\text{k}=2,3,4\right)\; \end{array}$$

The other parameter is **Pr**, which defines the proportion of all the SNPs in each of four normal distributions (Equation 5). The prior of **Pr** is drawn from Dirichlet distribution **Pr** ~ **Dirichlet**(**α**), with α = [1,1,1,1]. The conditional distribution of SNP effect on the proportion parameter **Pr** is $p\left(g_{i}|Pr\right)=Pr_{1}*N(0,0*\sigma_{g}^{2})+\;Pr_{2}*N(0,0.01*\sigma_{g}^{2})+\;Pr_{3}*N(0,0.1*\sigma_{g}^{2})+\;Pr_{4}*N(0,1*\sigma_{g}^{2})$ .

Bayesian models were fitted with the software GCTB (Zeng et al., 2018). For each model there were 25,000 iterations of the Gibbs chain with the first 5,000 iterations discarded as burn in. GEBV for validation clones (described below) were predicted as $GEBV=X\hat{g}$ . For other models including extended GBLUPs and ML approaches, phenotypic performance was predicted instead of GEBVs.

### 2.4 Machine learning methods

**Figure 1:**
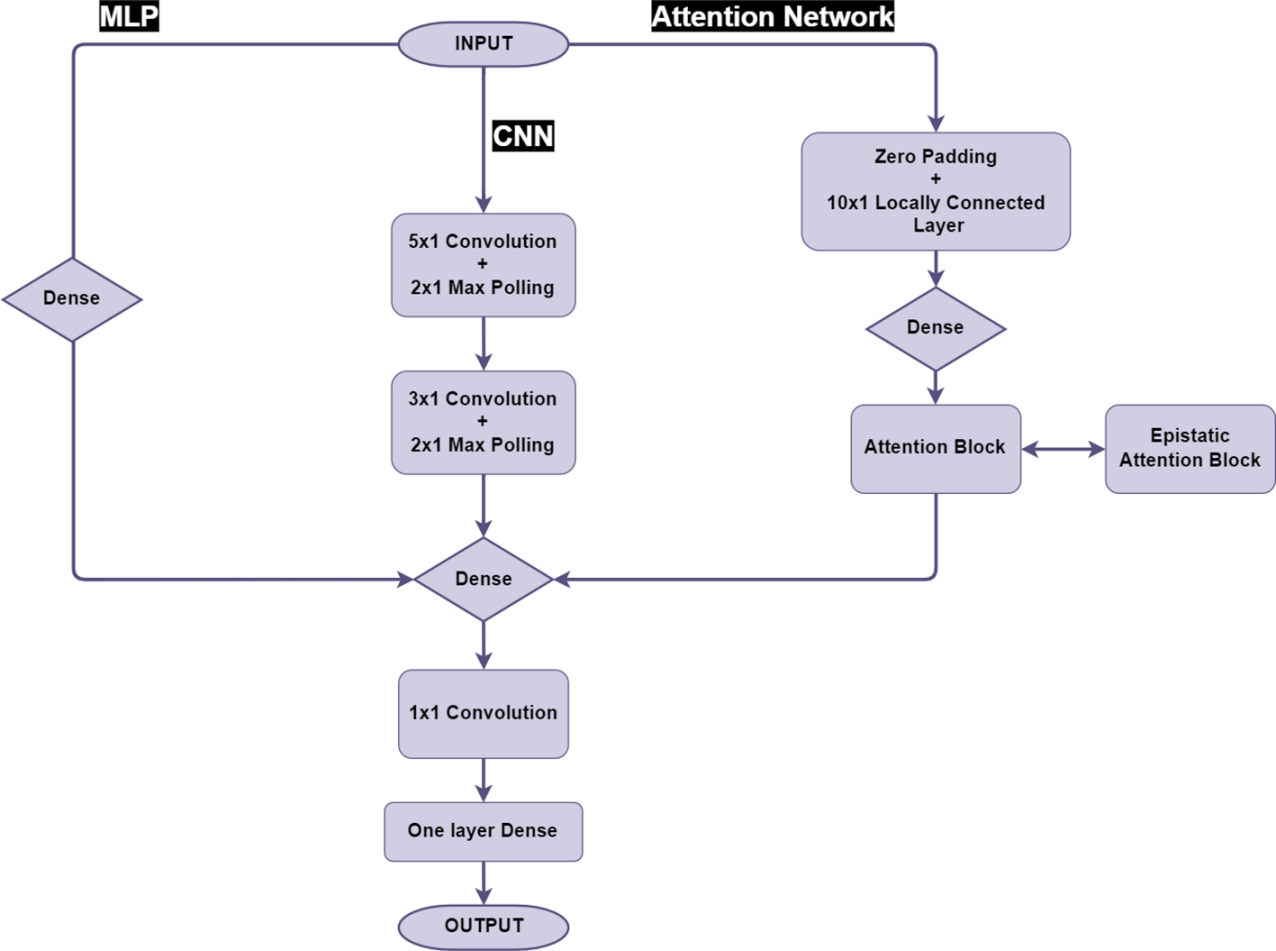
Model structure of MLP, CNN and Attention network.

**Figure 1** gives an overview of the model architecture of the neural networks. The RF, MLP and CNN for clonal prediction were implemented as described in Chen et al. (2023). For MLP and CNN the same layer structure was used at the end of the model, a 1x1 window, single-channel convolutional layer associated with a global average pooling layer to sum and average all the estimated effects as the final predictions. We also added one additional layer to partially enhance the non-linear predicting ability by using the sigmoid function to scale the 1x1 convolution outputs and feed the output into a one neuron fully connected layer, the output of both global average pooling layer and this single-neuron layer would be summed together as the predictions (**Figure 1**).

All the neural networks including MLP and CNN models were built using the Python packages TensorFlow (Version 2.9.1) (Abadi, 2016) and Keras (Chollet, 2015).

### 2.5 Attention network

The use of attention networks in this study was inspired by the major progress in the field of natural language prediction (Vaswani et al., 2017; He et al., 2020). Firstly a 16-channel (n) fully connected embedding layer was employed to obtain an expanded representation of SNP inputs by n trainable weights (and bias), given the standard form of attention value calculation (Equations 6, 7).


(6) $$\text{Q},\text{K},\text{V}={\text{X}}_{\text{SNP}}\cdot {\text{W}}_{\left(\text{Q},\text{K},\text{V}\right)}$$

(7) $$Attention\left(\text{Q},\text{K},\text{V}\right)=softmax\left(\frac{\text{Q}{\text{K}}^{\text{T}}}{\sqrt{d_{k}}}\right)\cdot \text{V}$$

According to our model structure, besides the initial SNP sequence (N,1), all other intermedia SNP information was multi-dimensional (N × n-dimensional), the embedded dimension was defined by weight shape of the previous layers and the choice of any n (channel number of the model structure, a hyper-parameter, 16 was used here) in different weight matrices were defined priorly as one of model structural hyper-parameters, as a standard strategy of neural network (Gardner and Dorling, 1998). Specifically in this model, the Query (Q), Key (K) and Value (V) represented encoded SNP information X passing through separated encoding matrices. *W* was the (n x n) matrix of encoding weights created by random initialization, each weight matrix belonging to encoding formula would be trained independently respect to encoding types (Q, K, V). The Attention values were a scalar value based on interactions with other SNPs and *d_k_* was the dimension of QKV array. The Softmax function represents the normalized probabilities for each input array as described in Vaswani et al. (2017). The attention calculation could be described in our context as all the SNPs have an effect of interactions with all the other SNPs. A single attention block was used in the attention network after the embedding section and was used to calculate the attention value for each input.

The attention blocks require calculation of a very large matrix (N x N) according to SNP numbers (N). This would easily exhaust GPU memory if we directly feed the raw SNP data to the model, and the same issues also exist if large N are processed by multiple neural layers (Gardner and Dorling, 1998). To ameliorate the memory issue, we used the strategy of locally connected layers to priorly compress and summarise the information from SNP array by merging them by N’-SNP segments that contain independent SNP weights, this could be described as segmental compression. The formula of merging SNP signals in single segment could be described as following equation (Equation 8):


(8) $$y_{j}=\;\sum_{i=1}^{N^{\prime }}w_{i}x_{i}$$

Where the *y_j_* is the output, as the SNP of jth segment, *w_i_* is the weight particular to SNP alleles (*x_i_*) in position i inside the segment, N’ was the previously chosen hyper-parameters for the segment length. It should be mentioned that unlike the convolutional kernel, segments in the locally connected layers would only calculate SNP signals for fixed SNP, every SNP would have its unique weights. Multiple channels were also applied into locally connected layers aimed to enhance the learning capacity. In summary, we selected a 32-channel (n=32), 10 SNP segment length (N’=10) locally connected layer before the attention encoding layer in our attention network, to compress the raw SNP sequence to one-tenth the length.

Furthermore, considering about the extremely long length of genetic SNP sequence (26K), the standard encoding learning with three (n x n) matrices may not be adaptable for capturing genome-wide epistasis. To overcome this limitation, we implemented a modification to the attention block used in our model, by adding an extra trainable weight matrix (N x N) as **W**_epi_ into the attention formula, in order to adjust the attention assigned to some SNP (Equation 9).


(9) $$Attention\left(\text{Q},\text{K},\text{V}\right)=softmax\left(\frac{\text{Q}{\text{K}}^{\text{T}}}{\sqrt{d_{k}}}\right)\cdot {\text{W}}_{epi}\cdot \;\text{V}$$

Before and after the attention block, a dense layer the same as the fully connected layer would be inserted to enhance the learning performance. The fully connected layer contains 32 neurons and manipulates the output for each channel.

All the DL models was built and trained by in-house Python program “GS_Composer” and currently available at GitHub repository (https://github.com/CCS-voidBird/GS_composer).

### 2.6 Hyper-parameter design

All the neural network models were trained with 30 epochs, using Mean squared error (MSE) as the loss function, initial learning rate 0.001 and 0.9 learning rate decay after trained by 6000 individuals cyclically. Due to the limitation of GPU memory, attention models using 26K SNP were trained with batch size 18. Parameters used in the attention network models are given in **Table 1**.

**Table 1:** Attention network parameters in one step (26k markers) and hybrid models (1000 markers).

Parameters	26K Markers	Hybrid models (1000 Markers)
*LCL Window*	10	1
*LCL Step*	10	1
*LCL Channel*	32	
*Batch Size*	18	64
*Learning rate (LR)*	0.001	
*LR decay step*	6000	
*LR decay rate*	0.9	

### 2.7 Hybrid models

The hybrid models had two steps; 1. marker selection, and 2. prediction.

We used either BayesR or GWAS to choose a subset of 1000 SNP. For BayesR the criteria was posterior probability of inclusion (PIP), with the 1000 SNP with the highest PIP selected, for GWAS the 1000 SNP with the lowest p-values of mixed linear model were used. These subsets were always chosen based on training sets only, information from validation set were never included when selecting the 1000 markers from a trained model (BayesR or GWAS). As a control we also evaluated a scenario where 1000 SNP were chosen at random within each cross-fold.

For the second step, a range of models were used to predict clonal performance, including GBLUP, extended GBLUP, BayesC, BayesR and Attention network. The structure of the attention network used in hybrid models was slightly modified from that described above including manipulating both window size and step of its locally connected layers into one, and training batch size would be increased to 64 because the reduced marker population would not exhaust calculation resource. **Table 1** describes details of the two stages of attention network modelling.

### 2.8 Random and PCA five-fold cross validation

**Figure 2:**
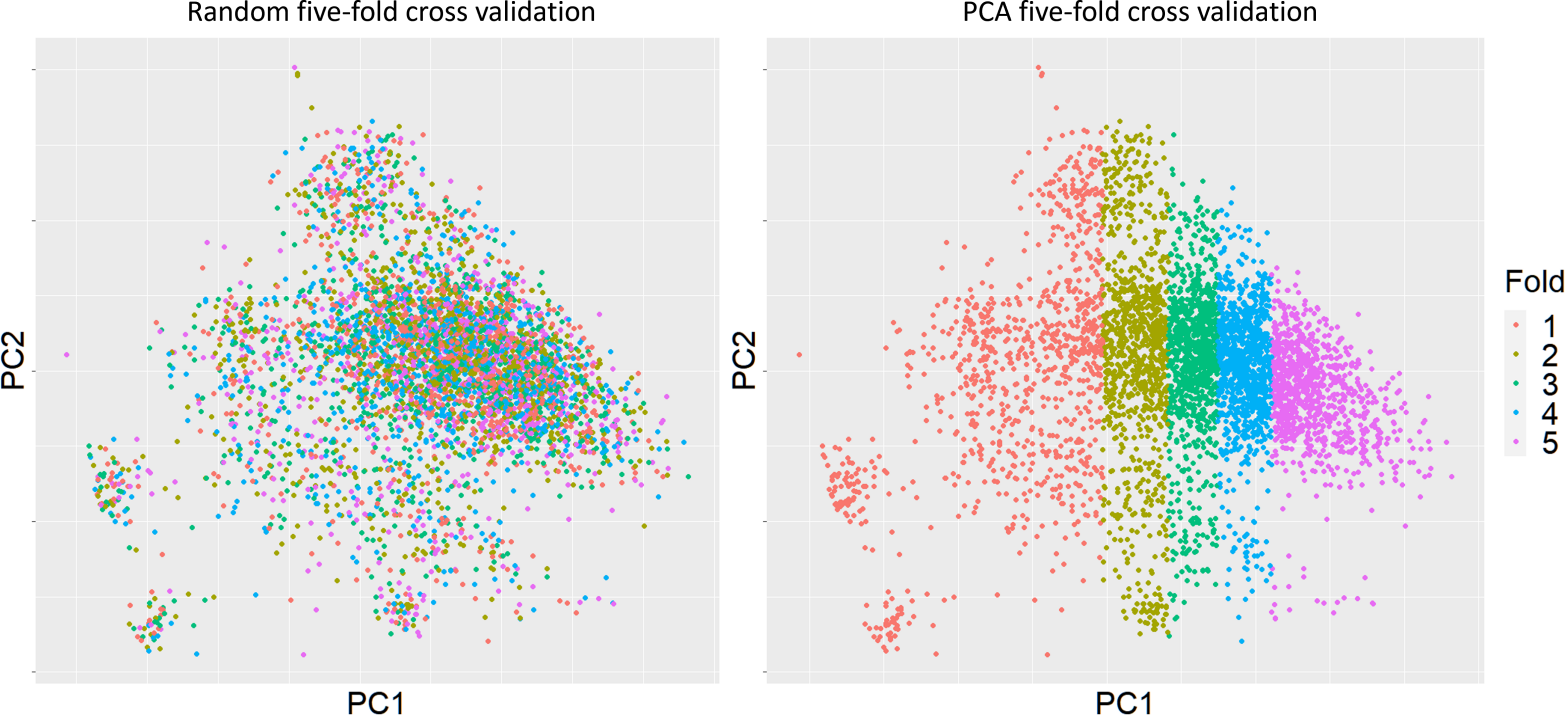
Clonal distributions of two types of five-fold cross validation based on the first principal component. The cross-validation subgroups were divided by using PC1 based on genomic relationships. Sugarcane clones for each fold were picked based on their PC1 values, each fold (20%) would contain almost same number of clones.

Two scenarios of cross-validation were applied during the prediction assessments. Random sampling was the first scenario, whereby five subsets of 20% of the data were sampled at random. Secondly, we performed PCA analysis of the genomic relationship among the 4702 sugarcane clones, and then PC1 used to separate sugarcane clones into training and validation five times, again with approximately 20% of the clones in each validation to keep training set sizes equal, **Figure 2**. This was termed “PCA five-fold cross validation” aimed to assess model performance when the validation set is less related to the training set. The distances between subsets were varying depend on orders. Fold 1 has relatively highest genomic variance comparing to other folds. The maximized genomic distance with PCA based splits was expected to bring difficulties to the prediction. Note that the assessment of hybrid models only used PCA cross validation.

## 3 Results

### 3.1 Variance components and heritability

**Figure 3:**
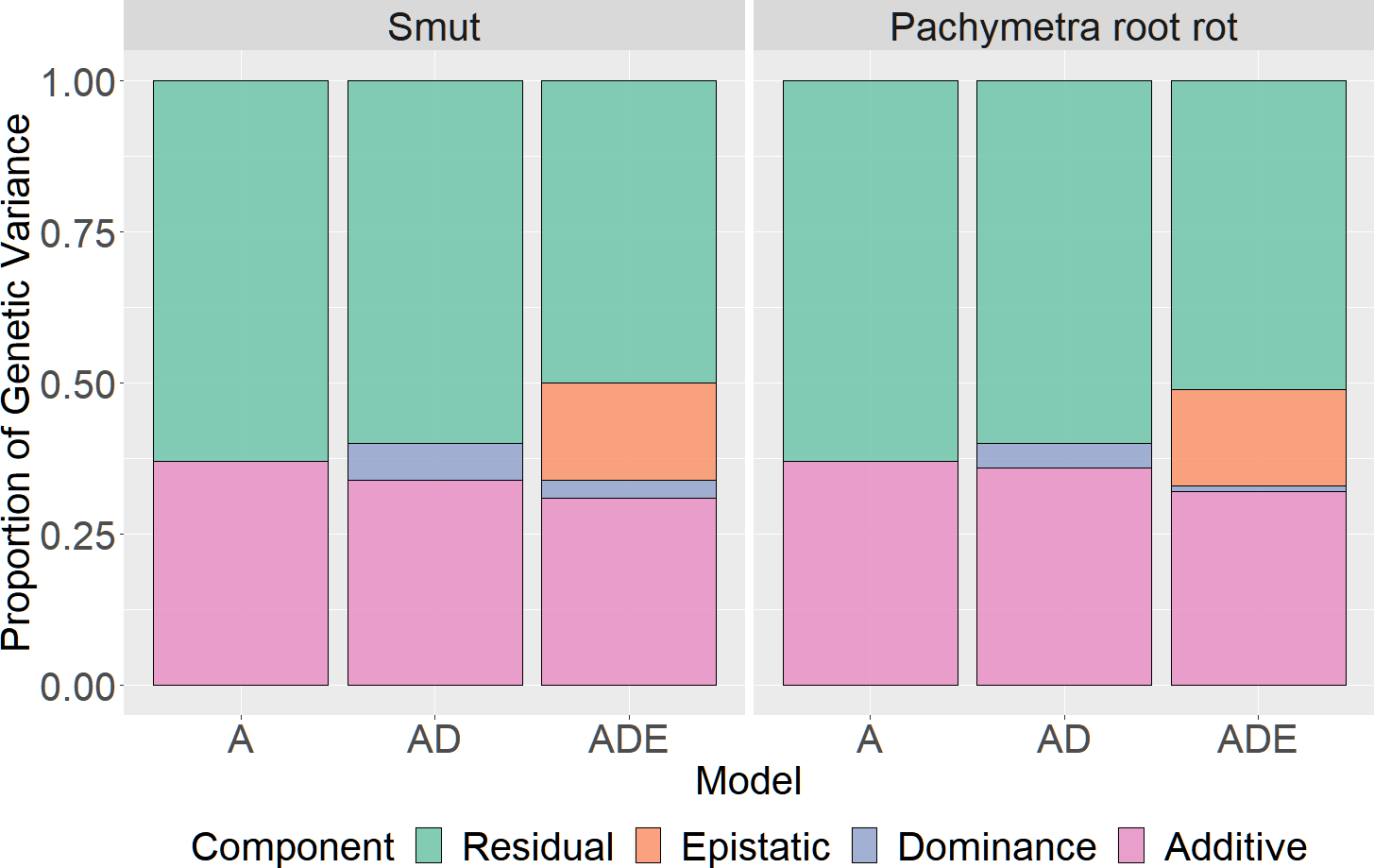
Proportion of phenotypic variance explained by additive, dominant and epistatic effects for smut and Pachymetra root rot scores. Variances were estimated by GREML and extended GREML models. The X axis was the GBLUP models: A - Additive GBLUP, AD - Additive dominant GBLUP and Additive, ADE – Additive, dominant and epistatic GBLUP.

Both disease traits had mainly additive variation, with only moderate to limited dominance or epistatic variation (**Figure 3**). According to the summary results of restricted maximum likelihood analysis (ADE GBLUP), 36% of the phenotypic variance was additive for smut, with only 4% for epistatic variance and limited dominance variance. For Pachymetra root rot, 34% of the phenotypic variance was additive, dominance effects accounted for 6% of the variance and epistatic variance was limited.

### 3.2 Performance of genomic prediction methods in cross-validation by random sampling and cross validation by PCA genomic distance sampling

Prediction accuracies from random cross-validation were generally higher than PCA five-fold cross validation. When PC1 was used to define reference and validation sets, prediction accuracy decreased by 16.1% for smut prediction and 14.2% for Pachymetra root rot prediction relative to accuracies in datasets using random cross validation when PC1 was used to define reference and validation sets.

**Figure 4:**
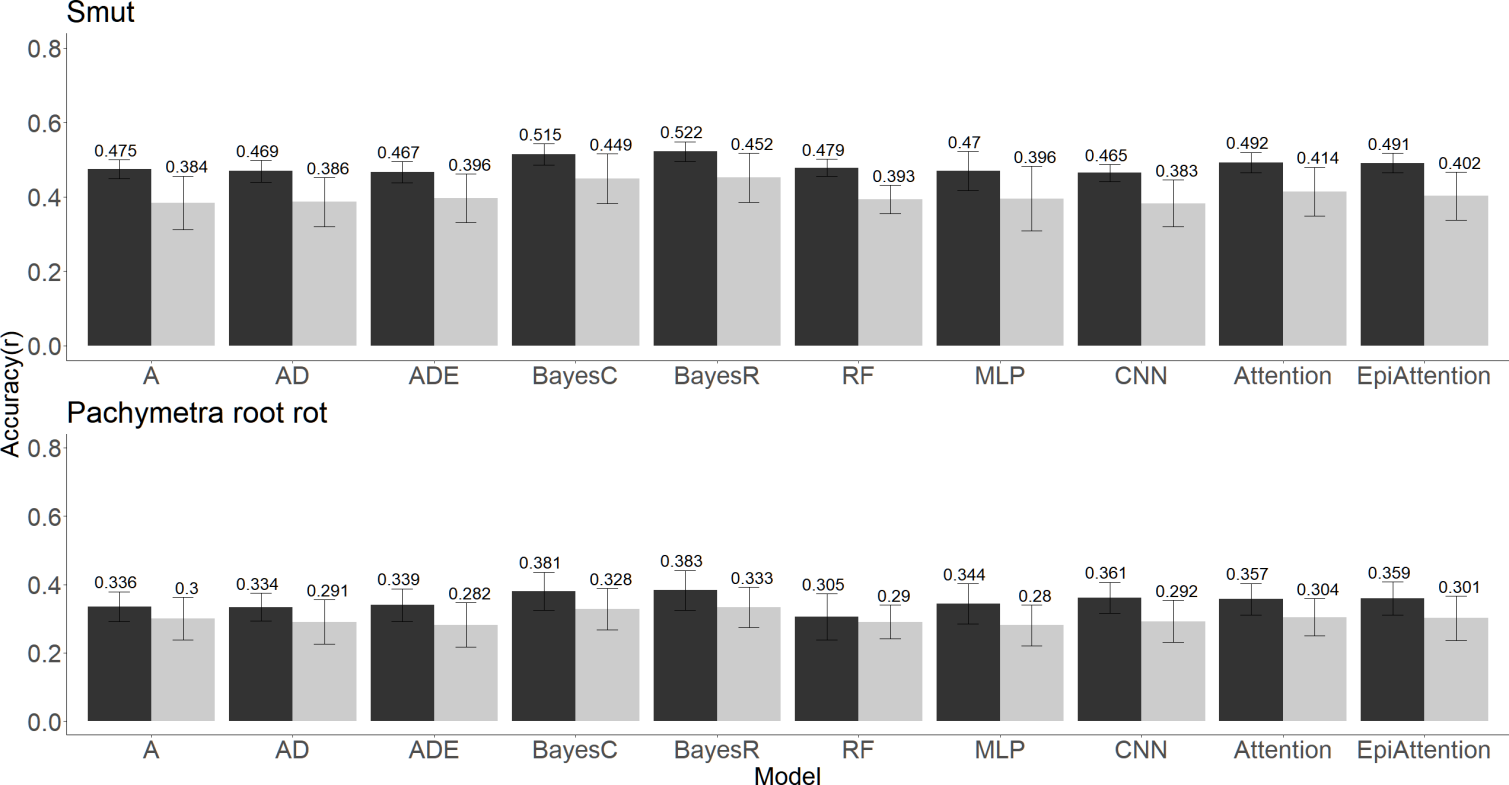
Clonal prediction accuracy among 10 models using all 26K markers under two types of five-fold cross-validations (random and PC1 separated) for smut (top panel) and Pachymetra root rot (bottom panel). The y axis is mean accuracy across five-fold cross-validation, measured as Pearson’s correlation. The error bars are the standard errors of the mean accuracy across the five folds. The X axis was the GBLUP models: A - Additive GBLUP, AD - Additive dominant GBLUP and Additive, ADE – Additive, dominant and epistatic GBLUP, Attention – Attention network, EpiAttention – Variant Attention network using additional epistatic matrix.3.3 Implications for breeding for disease resistance.

The prediction accuracy for Pachymetra root rot was lower than for smut regardless of the prediction method. Although differences between methods were modest, BayesC and BayesR gave the highest accuracies of prediction for both diseases across the ten algorithms. The attention network methods performed with second highest accuracies regardless of the cross-validation strategy used. (**Figure 4**). Meanwhile, The ML methods generally had the lowest mean square error (MSE) of prediction across the validation folds (**Supplementary Table 1**).

**Figure 5:**
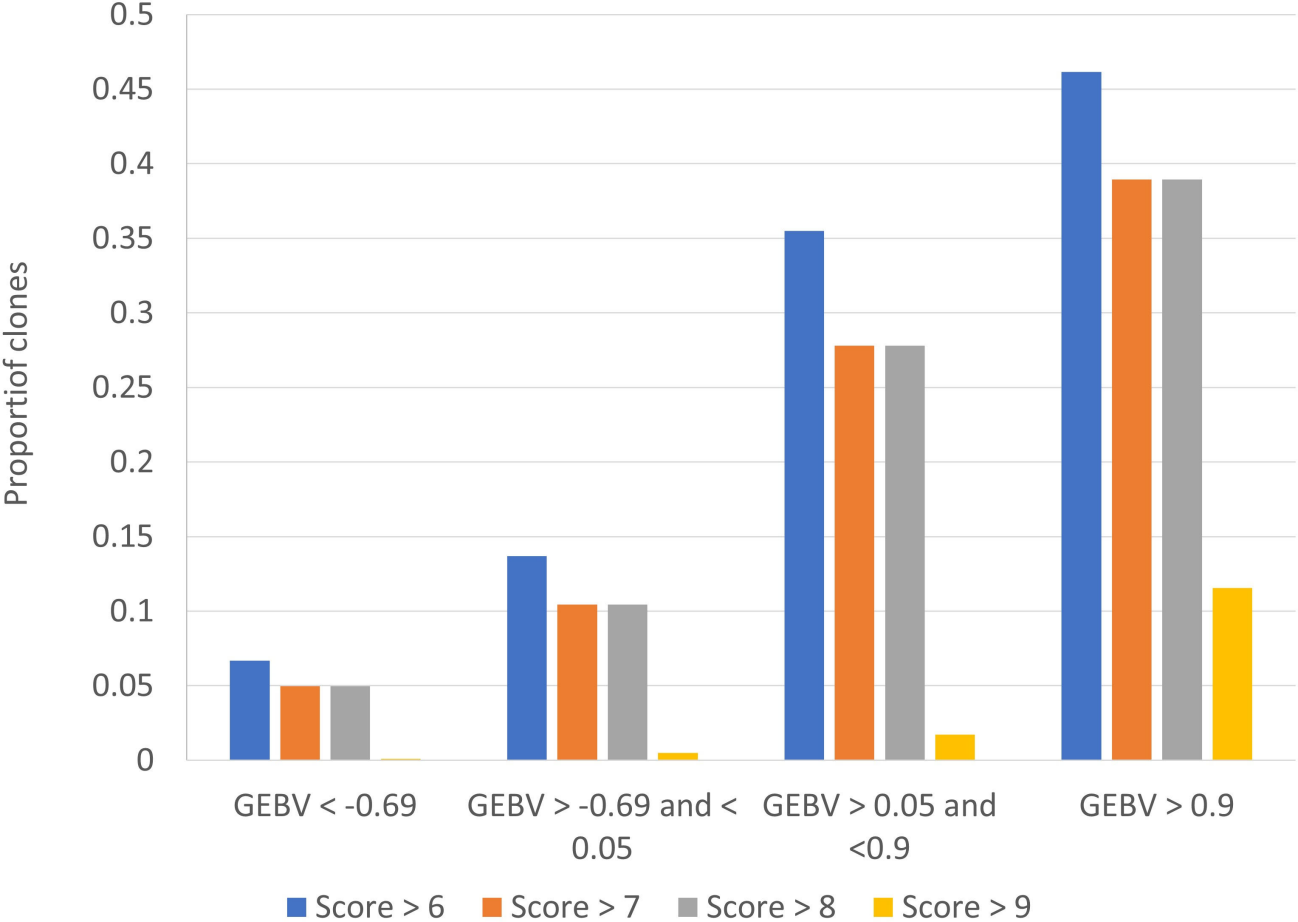
Probability of correctly assigning clones into extreme smut ratings, based on the clonal predictions (genomic estimated breeding value s, GEBV) from BayesR.

One of the challenges with disease resistance phenotypes in practise is that classification of intermediate types is less reliable than the tails of the distribution, and it is the tails that breeders are most interested in. To assess the ability of our genomic predictions to accurately identify clones with smut resistance in the tails of the distribution, we calculated the probability of correctly assigning clones into a category of< 4 rating, and alternatively the probability of correctly assigning into a bin of > 6, based on the GEBV from BayesR. We ranked the clones on their GEBV for smut, then looked at true smut ratings in the bottom ranked 25%, middle bottom 25%, middle top 25% and top (worst) 25%. **Figure 5** shows the percentage of clones in each band (quartiles) with a true rating of >6, >7, >8 and >9. The results indicate if the worst 75% of clones on smut GEBV are culled, and only the best 25% are taken forward in the breeding program, there is only a ~6% chance that a clone with a smut rating >6 (and only a 0.1% chance of a clone with smut rating of 9) will enter the breeding program. These results suggest breeders could use the clonal predictions to select for disease resistance with some confidence.

### 3.4 Genomic prediction accuracy from hybrid models

**Figure 6:**
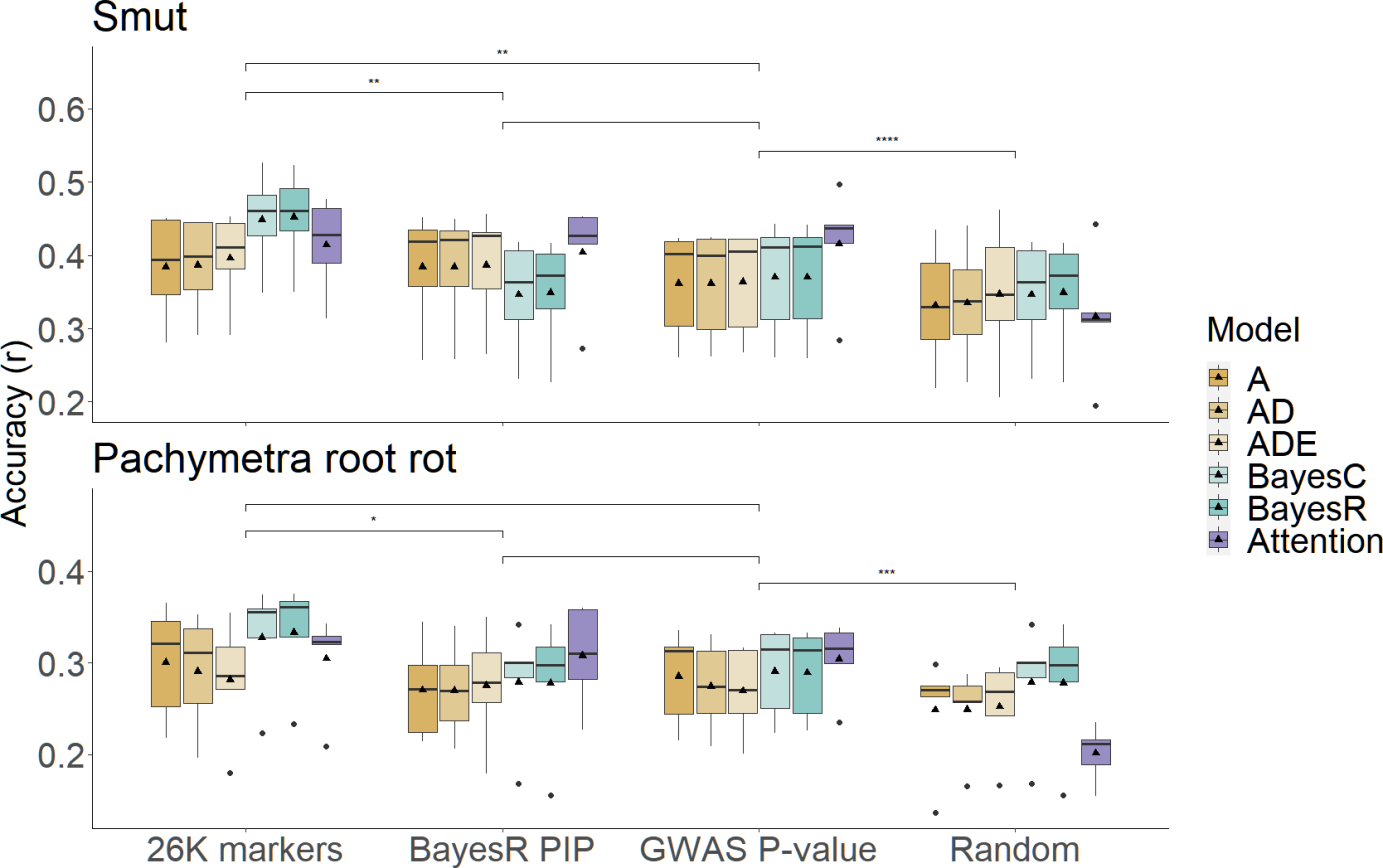
Comparison of prediction accuracies in scenarios using different sources of marker sets under PCA cross validation. X axis is scenarios including initial models using entire 26K marker set, hybrid models using high confidence marker subsets selected by BayesR PIP, GWAS P-value and random sampling. Boxes filled by different colours represent models/secondary models that make predictions in which bold dashes were median accuracies, triangles represented mean accuracies. Prediction accuracies were measured by Pearson’s correlation on the Y axis, results were separated into two disease traits. Significance within scenarios was assessed by pair-wised student-t test (*: p-value< 0.05, **: p-value< 0.01, ***: p-value< 0.001, ****: p-value< 0.0001).

To assess the effect of prior marker selection we compared the prediction accuracy of the full 26K marker set to the prediction accuracy obtained using only 1000 selected markers based on either the BayesR or GWAS results, or a random subset of markers of the same size. The combination of selecting 1000 markers followed by the attention network usually performed better than GBLUPs and ML models with all 26k markers, though the improvement was greater for smut than Pachymetra root rot. When the 1000 markers were chosen at random, performance of hybrid models was worse than one stage methods using entire 26K marker as predictors, demonstrating improvement in accuracy from the hybrid models was not just an artefact of using fewer markers in the prediction (**Figure 6**). The MSE of the hybrid approach with the attention network was much lower than other methods (**Supplementary Figure 3**) (**Supplementary Table 1**).

All hybrid models had significantly reduced compute time for the ML component compared to using 26K markers. In detail, the marker selection step of hybrid prediction would take the same time as the selected approaches running on the initial 26K data. The second step of using GBLUP and Bayes models as predicting approaches took about one minute using a 12-core computing server node. For the attention network, it would take about six minutes to finish an entire modelling session including training and validations, using a GPU (Nvidia V100) platform (**Table 2**).

**Table 2:** Compute time for hybrid model genomic prediction approaches.

Model	CPU cores	GPU requires	26k markers *	1000 markers **
**GBLUP**	12	No	~3 minutes	~1 minutes
Bayesian Alphabet***	12	No	~5 minutes	~1 minutes
GWAS****	12	No	~2 minutes	N/A
**RF**	12	No	<1 minute	<1 minute
**MLP**	1*****	Yes	~1 minute	~1 minute
**CNN**	1	Yes	~1 minute	~1 minute
**Attention network**	1	Yes	~1 hour	2-5 minutes

*: Modelling with 26086 markers.

**: Modelling with 1000 marker subsets.

***: BayesR model was also used for marker selection, as the first step of hybrid model.

****: GWAS was only applied for marker selection from initial 26K markers.

*****: By default, CPU in DL modelling process would only work on data I/O.

**Figure 7:**
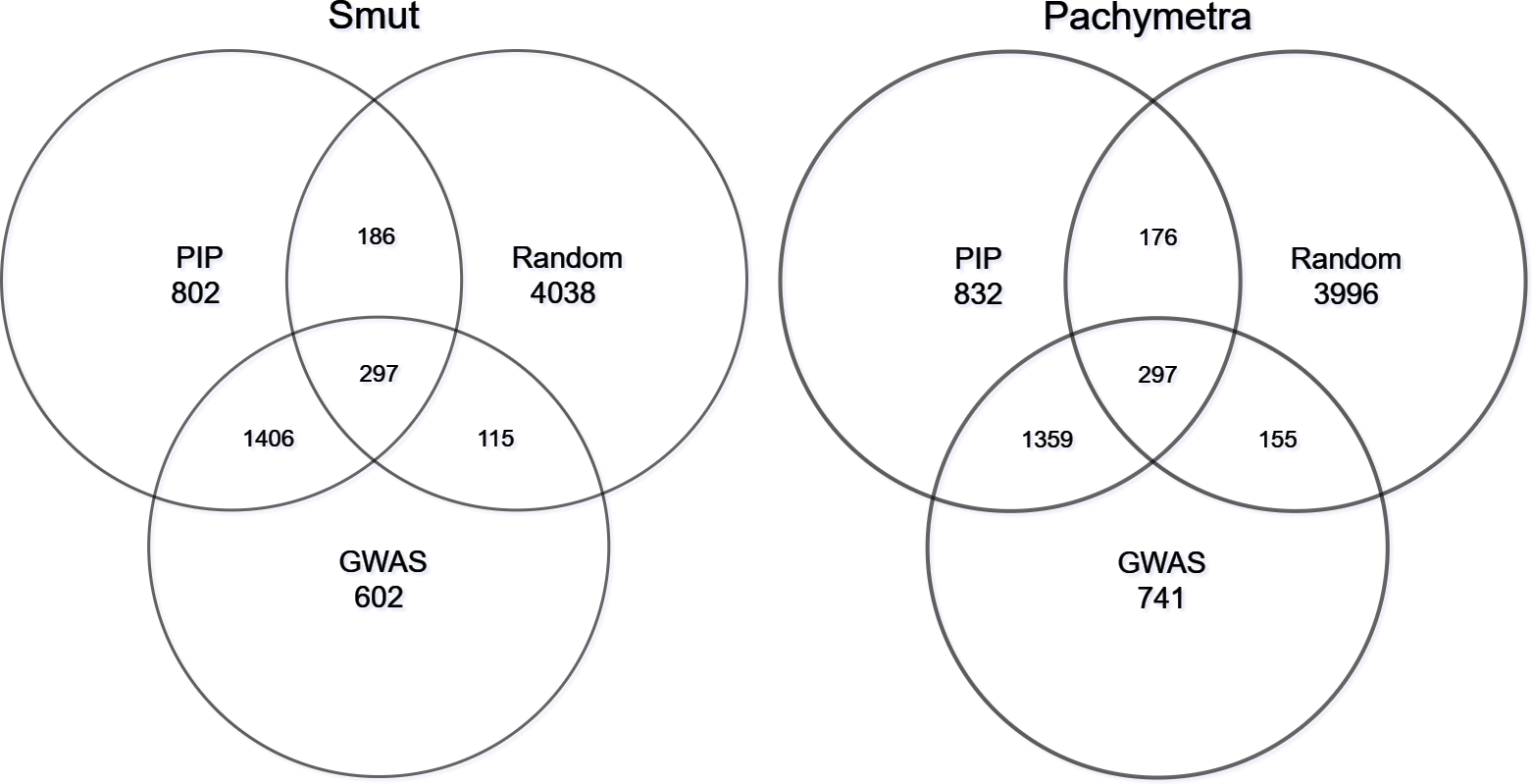
Proportion of markers shared within subsets. Two Venn plots represented marker intersects within different selection tools: Posterior inclusion probability (PIP) from BayesR, GWAS and random sampling.

We investigated the consistency of marker subsets generated by different selection procedures in the hybrid models, in which those markers were ranked and extracted from trained BayesR models (ranked by PIP), GWAS results (Ranked by -log10 p-value) and random sampling. Overall, for smut, across all folds 1703 markers were commonly discovered by BayesR and GWAS, 1656 markers were shared within Bayes and GWAS for Pachymetra root rot. In the random scenario only 297 and 155 markers were commonly shared for smut and Pachymetra root rot respectively (**Figure 7**). The fact that more markers are shared between BayesR PIP and GWAS than between either approach or random selection suggests that at least a proportion of the markers really are associated with large effects, although a lot of variability is induced by error.

## 4 Discussion

In this study we applied multiple genomic prediction approaches to predict resistance for two important diseases of sugarcane. Narrow sense heritabilities of both diseases were in good agreement with previous estimates [e.g., Croft and Berding (1994), Wu et al. (1988)]. Variation in both diseases had small contributions from epistatic and dominance effects.

For genomic prediction of clonal performance, hybrid methods combining marker selection using BayesR and attention network methods performed consistently well across both diseases and had the lower MSE.

### 4.1 Relative performance of genomic prediction methods for sugarcane disease is affected by genomic relationship.

BayesR was the method with the best performance across both traits and both validation scenarios, with the highest accuracy of clonal prediction for random cross validation for smut and Pachymetra root rot. The Epi-attention method also performed well, ranking amongst the top methods for all all-scenarios. The good performance of BayesR (and BayesC) in the PC1 cross validations may be because these allow for moderate to large effects, the marker-QTL associations identified persist across genetically more distant sub-sets of the population, as suggested and demonstrated by Kemper et al. (2018). We could speculate that the good performance of the attention and epi-attention methods, relative to other ML approaches, in PC1 cross validation is due to a related phenomenon – the attention methods do not “collapse” marker information to the same degree as the other methods, so markers with moderate to large effects can be captured in the output. We supposed that the lower accuracies in Pachymetra root rot predictions were probably caused by the poor data enrichment due to substantially lower number of clones phenotyped for Pachymetra

Recently, some authors have suggested that MSE of accuracy in cross-folds is an important criteria for assessing genomic prediction methods, as in practise crop and livestock breeders aim to reduce risk of future outcomes (e.g. varieties predicted to do well not performing as predicted) (Daetwyler et al., 2013). We observed that some of the ML methods, particularly attention networks, had lower MSE than GBLUP and Bayes methods, which may be an argument in their favour, though more research is required to understand how these methods achieve a lower MSE.

### 4.2 ML genomic prediction: insights and limitations

Comparing ML approaches used here, RF, MLP and CNN did not have consistently good performance across traits and validation strategies, consistent with previous research that implemented ML methods for genomic predictions (Heslot et al., 2012; Azodi et al., 2019; Abdollahi-Arpanahi et al., 2020; Mahood et al., 2020; Zingaretti et al., 2020; Chen et al., 2023). The attention network however did perform competitively with GBLUP models and Bayes models in most scenarios. This finding (and the finding that this method has lower MSE of prediction across cross-folds) would support the hypothesis that the attention network could be a useful alternative method for genomic prediction for complex traits. However, the structure of attention mechanism has its own limitations when applied to genomic prediction:

the predictors (usually diploid genotypes) in genomic prediction contained fewer categories (usually 0, 1, 2) than the attention network typically deals with which could significantly limit the ability of attention mechanism and cause fitting failure.
The large attention matrix formed from large markers sets (e.g., 26K markers) requires extremely large computational resources during the model training. Thus, we also suspected that the commonly used DL model structure: layer normalization or batch normalization, their availability would be significantly limited unless additional calculation resource could be invested, for example, performing parallel GPU modules. However, such implementation would significantly increase the computational cost and difficulty of implementation.

In our implementation of DL models including MLP, CNN and attention networks, we removed the normalization step in the model structures, and replaced the combination of ReLU & Batch-Normalization by Leaky ReLU to reduce the risk of gradient vanishing and neuron death. This resulted in less overfitting (Ioffe and Szegedy, 2015; Xu et al., 2015). This study also implemented and tested two modifications to the attention network which were aimed to solve issues mentioned above. First, the extended attention formula “EpiAttention” with additional trainable matrix did increase the prediction accuracy in the training set comparing to standard attention mechanism but couldn’t promise the advantages during the cross validation because the risk of overfitting was also increased. Secondly, a multi-channel locally connected layer synchronously mitigated exhaustion of memory in practice, and allowed information from each marker to be directly measured by multiple weights inside locally connected segments. The benefits of applying locally connected layers into neural network models have been previously implemented by Pook et al. (2020) and they observed positive results in accuracy of genomic prediction in Arabidopsis traits. However, specific benefits for adding locally connected layers into attention network models was not clearly verified. In addition, a recent study used another procedure to extended the marker diversities by replacing genotypes by genotypic allele frequency, such treatment received higher accuracy compared to models directly using genotypes (Jubair et al., 2021). All of these studies emphasized the necessity of increasing marker dimensions (e.g. using a weighting layer, associating with allele frequency) while applying attention mechanism into the genomic prediction. For instance to solve the low data dimension issue in genomic prediction that results from using discrete, diploidised genotypes, Hayes et al. (2023) suggested using marker haplotypes, which are much more variable than individual SNPs, as the input into attention networks, and this concept is supported by results from other studies in which annotated haplotype analysis (Liang et al., 2020) or prediction using haplotype blocks (Difabachew et al., 2023; Weber et al., 2023) was used.

Unlike linear mixed models, the neural network models require a solid learning epochs (e.g., 30) and have a high risk of fitting failure due to the random initialization, which is hard to resolve either through parameter tunning or training optimization. DL Models in this study were associated with a designed learning rate decay to reduce the risk of fitting failure caused by fixed learning rate, but still had the problems mentioned above because the tunning procedure was still quite limited, and has been determined with minor benefits in MLP and CNN by previous studies (Bellot et al., 2018; Abdollahi-Arpanahi et al., 2020; Han et al., 2021; Montesinos-López et al., 2021). For attention networks, general parameter tunning would be even more computationally expensive as the attention mechanism requires huge graphical calculations, although this could be at least partially resolved by using parallel computing across multiple GPU nodes.

### 4.3 Applying hybrid models for disease prediction

Our hypothesis that applying hybrid models in which the attention network was implemented on a subset of markers with moderate marginal effects for disease predictions seems to be at least partly confirmed. We speculate that this is because smaller number of markers used, allows more accurate estimation of marker interaction effects. A consistent marker set would give some confidence that the markers really were associated with mutations of larger effect.

A future direction worth investigating is incorporating Bayesian influence into neural networks, for example to directly select high confidence markers inside a neural network modelling. Some previous studies have verified that applying Bayesian influence into MLP and CNN with prior information could potentially benefit the prediction performance in the simulated dataset and real animal genomic prediction (Glória et al., 2016; Waldmann, 2018; Zhao et al., 2022).

## 5 Conclusion

This study implemented four main predicting algorithms (GBLUP, Bayesian alphabets, ML and hybrid methods) and a framework of hybrid models for predicting clonal performance for disease resistance in sugarcane. BayesR, BayesC and the attention network were the algorithms with the best performance. Attention network had higher accuracy and lower MSE than other ML methods. The modified attention network in a hybrid model with 1000 pre-selected markers had good accuracy across all scenarios, and very low MSE.

## Data availability statement

The data analyzed in this study is subject to the following licenses/restrictions: The data analyzed in this study is owned by Sugar Research Australia and the University of Queensland. It is possible that the data may be made available for specific cases on request. Requests to access these datasets should be directed to Prof Ben Hayes, b.hayes@uq.edu.au.

## Author contributions

CC: Conceptualization, Data curation, Formal analysis, Investigation, Methodology, Software, Validation, Visualization, Writing – original draft, Writing – review & editing. SB: Data curation, Formal analysis, Investigation, Methodology, Writing – review & editing, Resources. ER: Investigation, Methodology, Supervision, Writing – review & editing. OP: Investigation, Methodology, Supervision, Writing – review & editing. EDi: Investigation, Methodology, Supervision, Writing – review & editing. XW: Data curation, Investigation, Methodology, Resources, Writing – review & editing, Formal analysis, Funding acquisition. FA: Data curation, Investigation, Methodology, Resources, Writing – review & editing, Formal analysis, Project administration. EDe: Data curation, Investigation, Methodology, Resources, Writing – review & editing, Formal analysis. BH: Conceptualization, Data curation, Formal analysis, Funding acquisition, Investigation, Methodology, Project administration, Resources, Supervision, Visualization, Writing – review & editing.
