# Supplementary figures and images for: Genomic prediction for sugarcane diseases including hybrid Bayesian-machine learning approaches
Source: Front Plant Sci. 2024 May 1;15:1398903. doi: 10.3389/fpls.2024.1398903 (PMC11095127; doi:10.3389/fpls.2024.1398903)

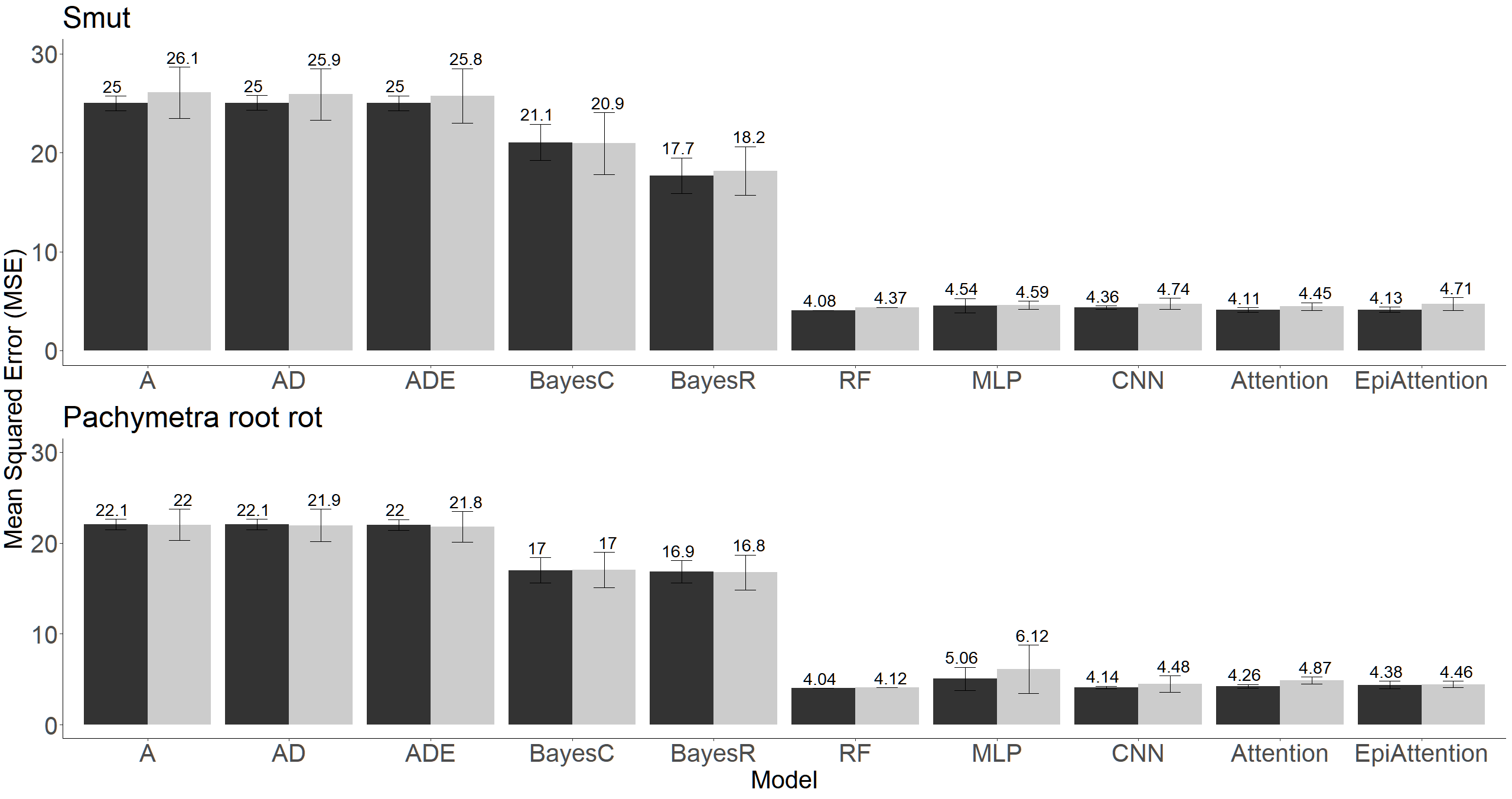

Supplement: Supplementary file 1 [file Image_1.jpeg]

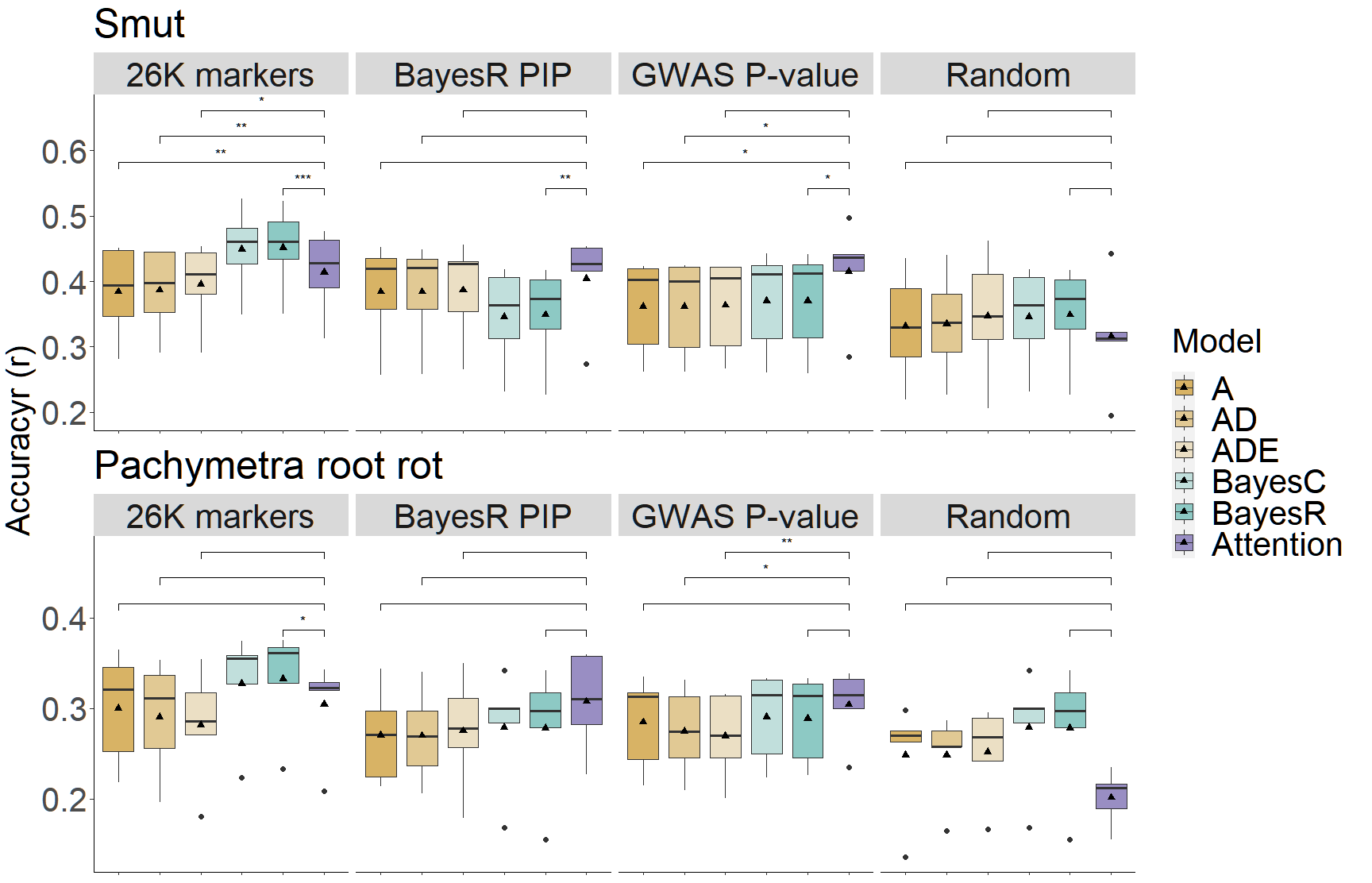

Supplement: Supplementary file 2 [file Image_2.jpeg]

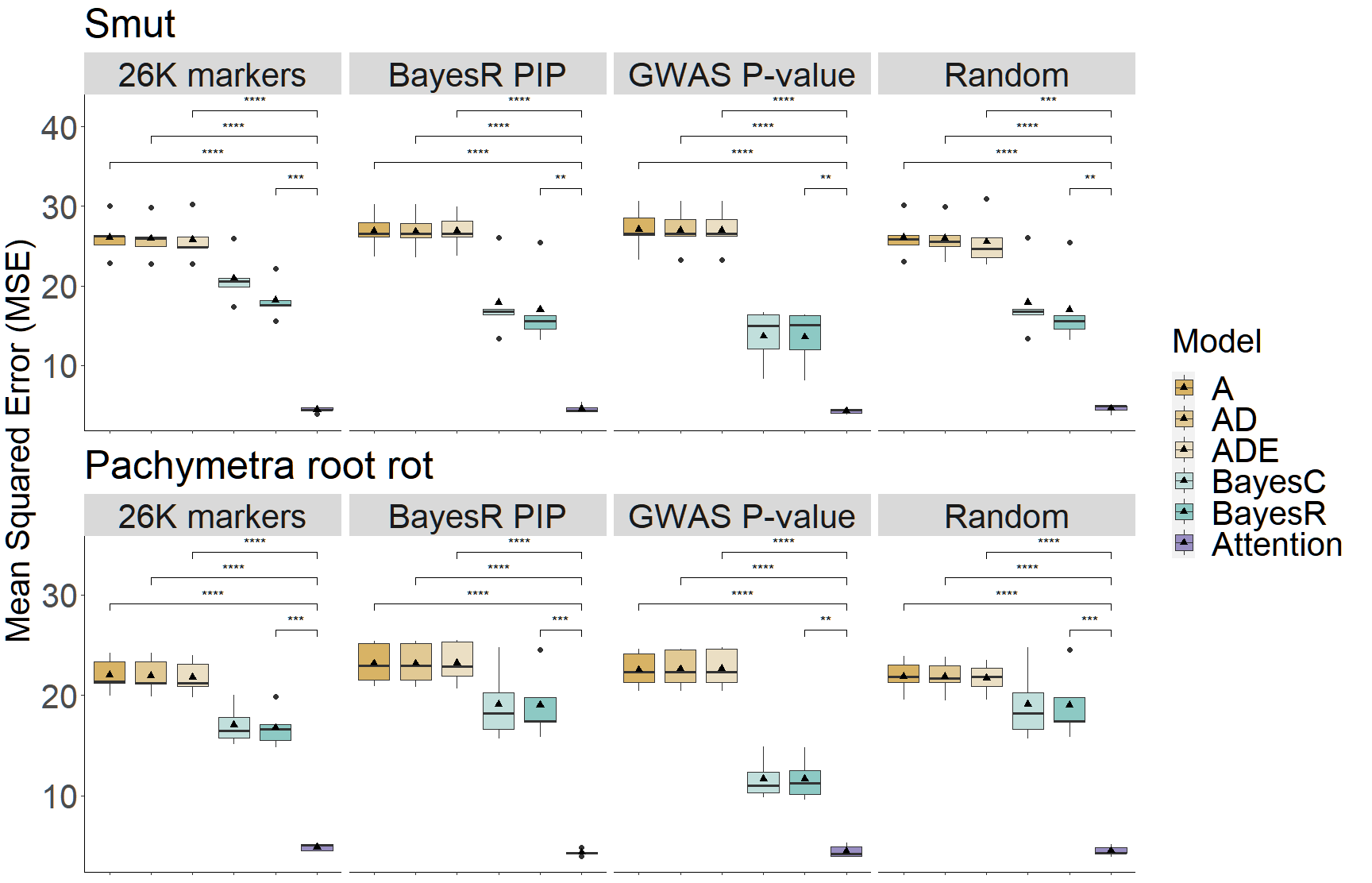

Supplement: Supplementary file 3 [file Image_3.jpeg]

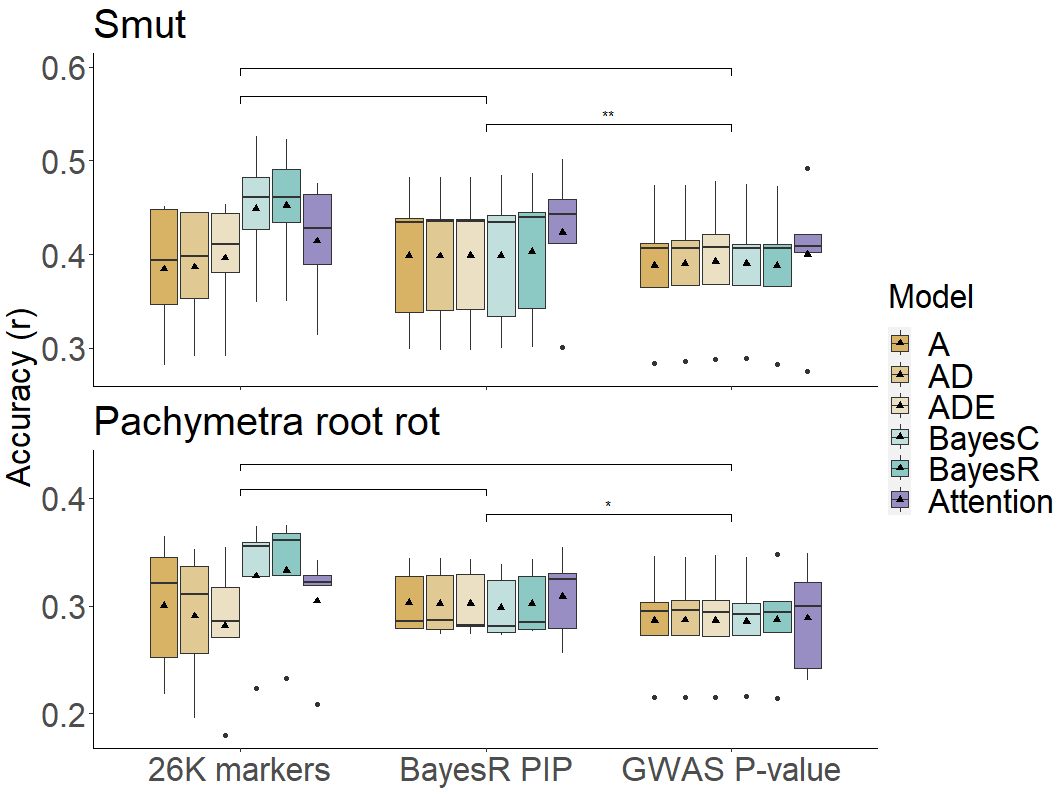

Supplement: Supplementary file 4 [file Image_4.jpeg]

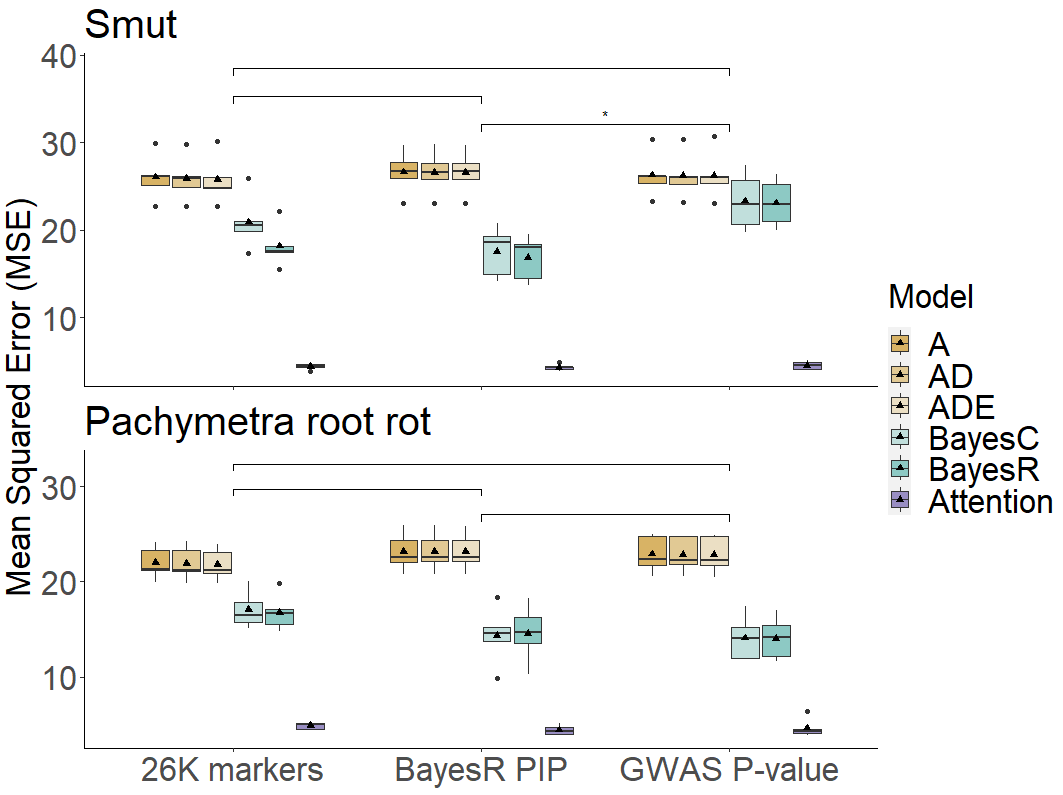

Supplement: Supplementary file 5 [file Image_5.jpeg]
